# Adaptations and early adoption of a family caregiver intervention in the Veterans Affairs Health Care System: A multimethod pragmatic approach for national scaling

**DOI:** 10.1111/1475-6773.14360

**Published:** 2024-08-01

**Authors:** Amanda C. Blok, Connor Drake, Kasey Decosimo, Leah L. Zullig, Jaime M. Hughes, Nina R. Sperber, Swetha Kota, Emily Franzosa, Cynthia J. Coffman, Megan Shepherd‐Banigan, Trisha Chadduck, Kelli D. Allen, Susan N. Hastings, Courtney H. Van Houtven

**Affiliations:** ^1^ Center for Clinical Management Research VA Ann Arbor Healthcare System Ann Arbor Michigan USA; ^2^ Department of Systems, Populations and Leadership University of Michigan School of Nursing Ann Arbor Michigan USA; ^3^ Durham VA Health Care System, Center of Innovation to Accelerate Discovery and Practice Transformation Durham North Carolina USA; ^4^ Department of Population Health Sciences Duke University School of Medicine Durham North Carolina USA; ^5^ Department of Implementation Science Wake Forest University School of Medicine Winston‐Salem North Carolina USA; ^6^ Section on Gerontology and Geriatric Medicine, Division of Internal Medicine Wake Forest University School of Medicine Winston‐Salem North Carolina USA; ^7^ James J. Peters VA Medical Center, Geriatric Research, Education and Clinical Center Bronx New York USA; ^8^ Icahn School of Medicine at Mount Sinai, Brookdale Department of Geriatrics and Palliative Medicine New York New York USA; ^9^ Department of Biostatistics and Bioinformatics Duke University School of Medicine Durham North Carolina USA; ^10^ Margolis Institute for Health Policy Duke University Durham North Carolina USA; ^11^ Veteran's Health Administration Central Office Washington DC USA; ^12^ Department of Medicine & Thurston Arthritis Research Center University of North Carolina at Chapel Hill Chapel Hill North Carolina USA; ^13^ Center for the Study of Aging and Human Development Duke University School of Medicine Durham North Carolina USA; ^14^ Division of Geriatrics, Department of Medicine Duke University School of Medicine Durham North Carolina USA

**Keywords:** adaptation, contextual analysis, implementation science, Veterans

## Abstract

**Objective:**

To examine the relationship between site‐level adaptation and early adoption of Caregivers Finding Important Resources, Support, and Training (FIRST) training during national implementation across diverse Veteran Health Administration (VA) medical centers.

**Data Sources and Study Setting:**

We enrolled and evaluated 25 VA medical centers (VAMCs). Along with administrative data on site characteristics, we examined site‐reported data on adaptations and intervention adoption, defined as ≥4 training classes delivered to ≥5 caregivers at 6 months from April through October 2022.

**Study Design:**

A type III hybrid implementation‐effectiveness cluster randomized controlled trial, randomized VAMCs 1:1 to receive foundational (low‐touch) implementation support (*n* = 12) or the addition of enhanced (high‐touch) implementation support (*n* = 13).

**Data Collection/Extraction Methods:**

At key implementation phases, VAMCs were asked to report adaptations including content, contextual modifications (format, setting, personnel, and population), and training of providers. We describe site‐level adaptations by arm and by organizational characteristics that included VAMC complexity level, staffing, rurality, and organizational readiness to change. We used qualitative comparative analysis to identify unique adaptations that contributed to intervention adoption at 6 months.

**Principal Finding**s**:**

VAMCs randomized to receive enhanced support reported slightly more adaptations than those randomized to foundational support. At 6 months, VAMCs with two or more adaptations adopted Caregivers FIRST at a higher rate than those with fewer adaptations (90% vs. 44%). Staffing adaptations (e.g., who delivered the intervention), format and content (e.g., modified delivery pace), and referring provider training were unique adaptations to adopting sites.

**Conclusions:**

Site‐level adaptations were diverse and occurred more frequently in sites with early adoption of Caregivers FIRST. Future research should identify best practices of supporting and monitoring intervention adaptation. Understanding the role of adaptation in early adoption success could assist other healthcare systems in implementing interventions for caregivers.

**What is known on this topic**

- Interventions developed and tested in research settings are rarely adopted in real‐world settings.
- Adaptation is often required to facilitate intervention implementation and scale; however, little is known on the nature of these adaptations and how they contribute to adoption and other implementation outcomes.

**What this study adds**

- This study uses novel survey methodologies for reporting intervention adaptations in real‐world practice settings in a proactive, theoretically sound, and streamlined manner.
- Overall, site‐level adaptations were diverse and occurred more frequently in sites with early adoption.
- Understanding the role of adaptation in adoption success could assist other healthcare systems to implement interventions supporting caregivers. Future research should identify best practices of supporting and monitoring intervention adaptation.

## 1 INTRODUCTION

In a fractured long‐term service and support system in the United States, 53 million family caregivers fill an essential role and provide unpaid annual economic contributions of over $600 billion.^1^ Despite evidence that education and support to caregivers can decrease psychological burden, improve caregiver depressive symptoms, and enhance health‐related quality of life, less than one in 10 caregivers report receiving the training they need^2^ and only a third access supportive services.^3^ The 2022 National Strategy to Support Family Caregivers promotes the spread of trainings and supports for caregivers; however, few caregivers have access to evidence‐based interventions.^2^ More research is needed to understand strategies for widespread dissemination and implementation of effective caregiver trainings, including how intervention adaptations can promote the translation of research into real‐world practice settings.^4^

Modifications to evidence‐based interventions are often necessary to ensure intervention‐setting fits in a specific population or delivery context (e.g., staffing, cultural factors, resources) where implemented. Adaptation is a type of intervention modification that is purposeful and proactive to enhance an intervention's feasibility, reach, fit, or effect on a population in a particular context.^5^ While some researchers are concerned the adaptations made by site‐level practitioners could have a negative impact on intervention fidelity and effectiveness, these adaptations are commonly necessary to improve fit across diverse local contexts and are appropriate for wider scaling. There is a need within implementation science to further understand site‐level adaptation of intervention delivery when considering scale‐up. Additional gaps include understanding optimal measurement and reporting of site‐level adaptations, the relationship between site context and adaptations, implementation strategies and adaptations, as well as the relationship between adaptation and implementation outcomes, such as adoption (i.e., the intention or action to deploy a novel practice or intervention).^6^, ^7^

We had a unique opportunity to assess intervention adaptation with the national rollout of an evidence‐based family caregiver skills training (Caregivers Finding Important Resources, Support, and Training (FIRST)) in the U.S. Veterans Affairs (VA) Health Care System among sites that did not meet adoption benchmarks within 6 months following the announcement of Caregivers FIRST as mandated within the VA Caregiver Support Program (CSP). This intervention was designed with flexibility that allowed for adaptation. Existing evidence on regional implementation of Caregivers FIRST suggests that adaptation may be critical given successful implementation requires changes to workflows, logistics, and practice patterns.^5^, ^8^, ^9^ We used Framework for Modification and Adaptations (FRAME), a reporting taxonomy for adaptation to understand motivations of, and reasons for, modification(s) in national scale‐up as well as several other key elements.^8^ FRAME classifications of adaptations included content, contextual modifications (format, setting, personnel, and population), and training of providers.

Using multiple data sources including staff surveys, VA administrative data, and intervention adoption data from the electronic health record (EHR) we addressed three research questions to increase our understanding of adaptation. (1) What types of adaptations did sites employ? We hypothesized that sites would report a variety of FRAME adaptations based on prior literature.^10^ (2) How are implementation strategies and site characteristics related to adaptation? We hypothesized that sites randomized to receive high‐touch implementation support (i.e., practice facilitation, collaborative problem‐solving) would have a greater number of reported adaptations than sites assigned to low‐touch implementation support, catalyzed by the level of engagement that high‐touch implementation support sites received.^11^ We also hypothesized that sites with higher levels of medical center complexity, caregiver program staff, and organizational readiness would report more adaptation compared to their counterparts.^12^ (3) What is the relationship between the number and types of adaptations made and adoption at 6 months? We hypothesized that sites reporting multiple adaptations would be more likely to adopt due to optimally tailoring the intervention to their site's and population's specific needs.^13^ This research will improve our understanding of the role of adaptation when interventions move into real‐world practice settings.

## 2 METHODS

Caregivers FIRST is an evidence‐based skills training delivered to friends or family members of Veterans that consists of four core classes delivered in a group setting.^13^ In 2022, Caregivers FIRST was announced for nationwide implementation such that VA medical centers (VAMCs) were required to deliver at least two caregiver group trainings in a fiscal year to meet VA CSP minimum performance standards.

As part of the Function and Independence Quality Enhancement Research Initiative, the study team enrolled 25 VAMCs over 12 months who had not reached adoption benchmarks, defined as either not adopting Caregivers FIRST or having “low enrollment” (defined in partnership with CSP as less than five caregivers trained). In this type III hybrid implementation‐effectiveness cluster randomized controlled trial, enrolled VAMCs were randomized 1:1 to receive foundational (low‐touch) implementation support (*N* = 12) or the addition of enhanced (high‐touch) implementation support with group‐based external facilitation (*N* = 13).^13^ Low‐touch implementation support was designed to be flexible and provided guidance on site‐specific adaptations and refinements as appropriate.^11^ High‐touch implementation support consisted of four facilitated phone calls over a period of 3 months that addressed adoption barriers and sharing of successful adoption strategies. The Standards for Reporting Implementation Studies (StaRI) checklist is reported elsewhere.^13^ This study was deemed IRB exempt.

### 2.1 Theoretical framework

To enable a framework‐informed and precise approach for reporting of site‐level adaptations, we used the FRAME taxonomy.^5^ We selected FRAME because it differentiates between cultural adaptations and those made for other reasons and because it assesses whether an adaptation was fidelity consistent. Fidelity‐consistent modifications are “those which do not alter core elements of treatment significantly enough to reduce adherence to a protocol and do not reduce ability to differentiate between treatments.”^14^ Aligned with the definitions put forth in FRAME, we considered content, contextual, and training adaptation classifications^5^ and operationalized them for our study (Box 1).

Box 1 **FRAME Taxonomy Operationalized Definitions of Adaptations**

*Content*. “Changes made to the intervention procedures, materials or delivery.” There are seven possible levels these changes could be made. These include: individual recipient, cohort, population, provider/facilitator, unit, hospital/organization, or network/community level.

*Contextual modifications*. “Changes made to delivery of the same program content, but with modifications to the format or channel, the setting or location which the overall intervention is delivered, or the personnel who deliver the intervention.”^15^ These include four subclassifications: format (the format, frequency, or channel of delivery was changed), setting (setting or location the intervention is being delivered was changed), personnel (situated within the CSP or amount delivering intervention was changed), and population (target population was changed).

*Training and evaluation*. “Changes made to the procedures for training personnel or evaluating the program…occur ‘behind the scenes’ and do not necessarily impact intervention content or the context of delivery.”^15^ These may include reaching out across service lines or communicating/marketing the intervention to other personnel or providers.^5^

### 2.2 Setting and participants

In the first 6 months of the minimum performance standard period (October–March 2022), VAMCs were eligible for study recruitment if they had not adopted Caregivers FIRST or had “low enrollment.” Sixty‐seven sites had met implementation benchmarks in the first 6 months out of 142 total sites. As such, the remainder, a total of 75 sites, were eligible for recruitment and prioritized by VA service region, site complexity level, implementation activity (no Caregivers FIRST activity vs. adopted but with “low enrollment”), and CSP staffing capacity.^13^ Of this list, at least two sites per VA service region (18 total) were systematically approached, prioritizing two site‐level factors: lower complexity, meaning VAMCs are typically rural and may have less staffing to support caregiver programming^16^, ^17^ and lowest implementation activity. The study team presented Caregivers FIRST on VA regional and site‐level calls and followed up with individual medical centers to confirm study eligibility and secure a signed participation agreement that exhibited facility leadership support and willingness to deliver Caregivers FIRST within 6 months. The recruitment goal was 24 sites. Of the 75 initial eligible sites approached, 25 enrolled (33%).

### 2.3 Data collection

Based on FRAME, we developed a site‐level survey with discreet and open‐ended questions that inquired about site‐initiated adaptations in content, contextual modifications (format, setting, personnel, and population), and training of providers based upon prior work.^13^ The baseline survey assessed how each site had adapted (or planned to adapt if they had not launched) Caregivers FIRST. The 6‐month survey, which was collected after randomized sites received high‐touch implementation support, assessed if each site had made any changes since the baseline assessment, using FRAME to design survey questions, such as when the adaptation was made, if it was planned, who participated in the decision‐making, and the goal behind making the adaptation. See Appendix for survey instruments.

From April 2022–April 2023, site‐level point(s) of contact (POCs) were asked to voluntarily participate in the survey, which captured adaptations at baseline and 6 months. Surveys were administered electronically via VA REDCap (Research Electronic Data Capture)^18^ by sending an email with two weekly reminders to unique POCs representing the 25 participating sites. POCs at each site (with 36% having co‐leads) were responsible for leading Caregivers FIRST delivery. Of the POCs, all were CSP staff, with 12 (30%) program managers, 25 social workers (63%), and two nurses (5%). For the baseline survey, 31 POCs representing all 25 sites completed the survey and for the 6‐month follow up, 23 POCs representing 20 sites completed the survey (Appendix Figure S1).

### 2.4 Implementation support

**FIGURE 1:**
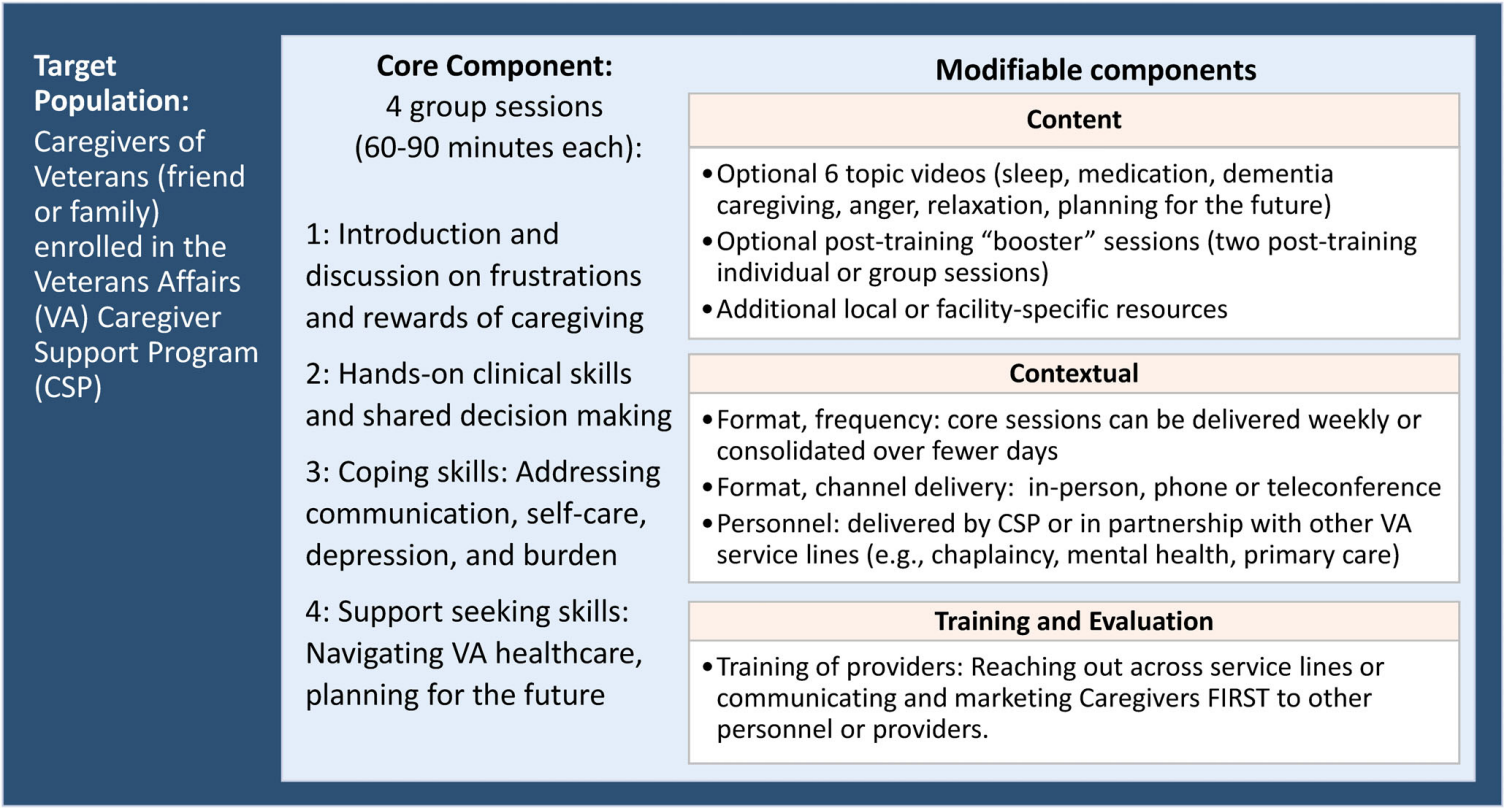
Core and modifiable components of Caregivers Finding Important Resources, Support, and Training (FIRST).

All 25 enrolled sites received foundational low‐touch implementation support^11^ comprised of self‐guided strategies, toolkits, the Caregivers FIRST data dashboard, and a learning collaborative based on the Replicating Effective Programs (REP) framework.^13^, ^19^ Beyond this, enrolled sites were randomized 1:1 to low‐touch only or high‐touch implementation support. The low‐touch implementation support arm received an initial “welcome call” providing an overview of the available support tools, including a toolkit of recorded training webinars that specified the core components of Caregivers FIRST and provided guidance for local customization options for intervention adaptation (Figure 1). The high‐touch implementation support arm received four group calls with tailored facilitation led by the study team addressing key implementation barriers. We conducted a needs assessment for each high‐touch site to identify common implementation barriers and then tailored the content of the calls to address these. Additional details are published elsewhere.^13^

### 2.5 Measures

Our primary outcome was early adoption of Caregivers FIRST defined as, delivering the four classes to a minimum of five caregivers within 6 months. Administrative information for each site was sourced from program documentation templates from the national EHR (Box 2). Explanatory variables of interest were facility complexity, staffing, demand for caregiver support services, geographic region, organizational readiness for implementing change,^17^, ^20^ organizational resilience,^21^ and implementation climate scale.^22^ These were selected because they align with the overarching conceptual model of the study's implementation intensification framework^13^ and are factors associated with implementation success.

#### 2.5.1 Organizational characteristics

We assessed organizational characteristics based on administrative data (e.g., facility complexity, etc.), site‐reported baseline survey (e.g., organizational readiness for implementing change), and program data (e.g., staffing, demand, etc.). These measures were selected because these are factors that are anticipated to impact intervention adoption.

Box 2 **Organizational Characteristic Measures**

*Facility complexity*. Ratings categorize each VAMC based on levels with level 1 being most complex to level 3 being the lowest level of complexity. Complexity levels are determined by the following factors: patient volume, patient risk, physician specialists, and teaching and research.^16^ Since high complexity medical centers serve the largest volume of patients, they may also serve more caregivers and have more staff to support caregiver programming.^17^

*Staffing*. Staffing includes the number of CSP full‐time staff hired or planned to be hired for each facility. Internal programmatic data for fiscal year 2021.

*Demand for caregiver support services*. Service demand includes internal programmatic data from the CSP of unique applications submitted by the Veteran and their caregiver for the Program of Comprehensive Assistance for Family Caregivers (PCAFC). The number of applications could be a proxy for CSP volume or demand at each facility.

*Geographic region*. Geographic regions (West, Midwest, Northeast, and South) are based on the US Census Bureau regions.^23^

*Organizational Readiness for Implementing Change (ORIC)*: At study baseline, we used a 9‐item scale assessing organizational members' shared resolve to implement a change (change commitment) and shared belief in their collective ability to make the change (change efficacy).^17^, ^20^ Each item includes a Likert scale from 1 (Disagree) to 5 (Agree) and the score is the average of all respondents from each site (mean of multiple responses). Recent findings suggest that ORIC scores were associated with timely adoption of Caregivers FIRST.^17^

*Organizational Resilience*: Emerging from the field of disaster preparedness, this validated measure assesses the capacity of a broader organization to anticipate, prepare for, respond, and adapt to incremental changes and sudden disruptions.^21^ At baseline, we calculated this as the mean of the sum of the eight survey questions for each site.

*Implementation Climate Scale*: Captures six dimensions of the organizational context that indicate to employees the extent to which their organization prioritizes and values the successful implementation of Caregivers FIRST.^22^ At baseline, we calculated this by taking the mean of responses for questions within each scale, and then taking the mean of all six scale scores.

### 2.6 Analyses

We used descriptive analysis to describe organizational characteristics of the sites. We also compared differences in characteristics of those who completed the adaptation survey at 6 months (*N* = 20) from those who did not (*N* = 5). We report the prevalence of adaptations and their classifications among sites delivering the intervention. Then, using a directed qualitative content analysis (DQICA) of open‐ended survey questions, we describe the nature, goals, and reasons prompting adaptations. We examined these adaptations using a rapid qualitative analysis of our open‐ended data. We adapted this methodology for our qualitative data analysis, informing our matrix structure of adaptation values by site. Two separate coders—implementation scientists with scientific backgrounds in nursing (ACB) and public health (CD)—applied the adaptation survey data to the matrix separately. After comparing matrices, differences were reconciled, and a combined matrix was finalized. Additional experts in health services research (CVH, EF, NS) reviewed the data, matrix, and summaries for rigor.

We also examined the relationship between implementation strategies, site‐level organizational characteristics, and site‐level adaptations. Using two‐way and three‐way tables, we described overall site organizational characteristics and adaptations, then described these by arm (high‐touch and low‐touch implementation support).

We explored the relationship between adaptations and early adoption of the intervention by site at 6 months. Early adoption was defined as ≥4 training classes delivered to ≥5 caregivers each at 6 months. For those who were early adopters, we describe those who implemented 5–8 training classes as moderate adopters and those who implemented >8 training classes as high adopters. We describe differences in rates of early adoption for sites that performed multiple adaptations versus those who performed one or less.

## 3 RESULTS

### 3.1 Descriptive data

We enrolled and evaluated 25 VAMCs across the United States (10 South, seven Northeast, five Midwest, three West) over 12 months. Just over half of sites enrolled had a high medical complexity classification and one fifth were in a rural location. Baseline characteristics of these sites by implementation arm are reported (Appendix Table S1). Sites in the low‐touch arm are more likely to be from the South, have a higher facility complexity, and slightly more experience implementing evidence‐based practices. Sites completed an adaptation survey at baseline (*N* = 25) and 6 months (*N* = 20) which were included for this analysis (Table 1). The five sites that had staff complete the baseline survey but did not complete the 6‐month survey were more likely to be at high complexity medical center and have higher demand for caregiver services. These sites did not receive enhanced implementation support yet reported a higher amount of previous implementation experience. These five sites also had slightly less CSP staff than those who did complete the adaptations survey. Organizational resilience and Implementation Climate Scale did not appear related to adaptation survey completion.

**TABLE 1:** Baseline organizational characteristics by adaptation survey completion.

	Overall (*N* = 25)	Adaptation survey completion	
		Sites completed survey (*N* = 20)	Sites did not complete survey (*N* = 5)
	*N* (%)	*N* (%)	*N* (%)
Facility complexity, two or higher	13 (52)	9 (45)	4 (80)
Received high‐touch implementation support	13 (52)	12 (60)	1 (20)
Rural	5 (20)	4 (20)	1 (20)
Location			
South	10 (40)	7 (35)	3 (60)
Northeast	7 (28)	5 (25)	2 (40)
Midwest	5 (20)	5 (25)	0 (0)
West	3 (12)	3 (15)	0 (0)
Site's experience implementing evidence‐based practices^a^			
Quite a lot, a fair bit, or some	22 (88)	18 (90)	4 (80)
Very little or none	0 (0)	0 (0)	0 (0)
Don't know/Missing	3 (12)	2 (10)	1 (20)

	Mean (SD)	Mean (SD)	Mean (SD)
CSP staffing	11 (4)	11 (4)	10 (2)
PCAFC unique applications	358.4 (232.1)	342.7 (247.6)	421.4 (138.8)
Organizational readiness	4.5 (0.3)	4.5 (0.3)	4.5 (0.1)
Organizational resilience	24.5 (2.9)	24.5 (3.1)	24.8 (2.1)
Implementation climate	2.1 (0.5)	2.0 (0.5)	2.3 (0.4)

Abbreviations: CSP, Caregiver Support Program; ICS, Implementation Climate Scale; ORIC, Organizational Readiness for Implementing Change; PCAFC, Program of Comprehensive Assistance for Family Caregivers; SD, standard deviation.

^a^ Facility experience implementing evidence‐based practices is self‐reported and was collected by each site point of contact at study intake.

### 3.2 Main results

#### 3.2.1 Documented adaptations

The majority of sites reported adaptations to the intervention (*N* = 16/20; 80%), with over half reporting ≥2 adaptations (*N* = 11/20; 55%) and over a third reporting adaptations in multiple FRAME classifications (*N* = 7/20; 35%) (Table 2). Some sites made multiple adaptations within specific FRAME adaptation classifications (*N* = 8/20; 40%), including in areas of personnel (*N* = 5), format (*N* = 2), and content (*N* = 1). Most adaptations were around recruiting (*N* = 6 adaptations) and delivering (*N* = 6) the intervention, as well as exploration of service line collaboration (*N* = 3). Sites also adapted the format of the classes, including adjusting their pace (*N* = 6) and how they were delivered (e.g., remote vs. in‐person) (*N* = 4).

**TABLE 2:** Types of adaptations employed.

		Implementation support	
	Sites implementing adaptations overall (*N* = 20)	High‐touch implementation support (*N* = 12)	Low‐touch implementation support (*N* = 8)
Amount and diversity of adaptations	*N* sites (%)^a^	*N* sites (%)	*N* sites (%)
Two or greater adaptations	11 (55)	7 (58.3)	4 (50)
Two or more classifications	7 (35)	4 (33.3)	3 (37.5)

Adaptation classifications	*N* sites (%)^a^	*N* adaptations^b^	*N* sites (%)	*N* adaptations	*N* sites (%)	*N* adaptations
Personnel	8 (40.0)	15	4 (33.3)	9	4 (50.0)	5
Program Recruitment	6 (30.0)	6	4 (33.3)	4	2 (25.0)	2
Program Delivery	6 (30.0)	6	4 (33.3)	4	1 (12.5)	1
Cross‐Service Line Collaboration	3 (15.0)	3	1 (8.3)	1	2 (25.0)	2
Format	7 (35.0)	10	5 (41.7)	8	2 (25.0)	2
Delivery	3 (15.0)	4	2 (16.7)	3	1 (12.5)	1
Pace	6 (30.0)	6	5 (41.7)	5	1 (12.5)	1
Training	3 (15.0)	3	2 (16.7)	2	1 (12.5)	1
Content	4 (20.0)	5	3 (25.0)	3	1 (12.5)	2
Optional material	2 (10.0)	2	1 (8.3)	1	1 (12.5)	1
Add class	1 (5.0)	1	0	0	1 (12.5)	1
Remove material	2 (10.0)	2	2 (16.7)	2	0 (0)	0
Population	2 (10.0)	2	2 (16.7)	2	0 (0)	0

^a^ Sites may make multiple adaptations within a classification, resulting in the number of sites in subclassifications not adding up to the number of sites making adaptations in a classification. All percentages are calculated using the column site sample size as the denominator (e.g., *N* = 20 sites overall, or *N* = 12 high‐touch implementation sites, or *N* = 8 low‐touch implementation sites).

^b^ Adaptations made in subclassifications of an adaptation classification (either Personnel, Format, Training Content or Population) all do add or sum up to the classification total.

##### Adaptations by implementation strategy

While sites with high‐touch implementation support were slightly more likely to employ ≥2 adaptations (58.3% vs. 50.0%), they were not more likely to have adaptations in multiple FRAME classifications. Sites with high‐touch implementation support were more likely to make adaptations to intervention format (e.g., changes to the delivery, pace, and content of the caregiver classes) than sites with low‐touch support (42% with eight changes vs. 25% with two changes) and only high‐touch sites made adaptations to the population of caregivers from which they recruited. Personnel adaptations occurred more frequently in sites with low‐touch support (50% vs. 33% in high‐touch sites), where sites with low‐touch support more often explored collaborations across service lines for program recruitment and delivery.

##### Descriptions of adaptations

From our qualitative analysis, we describe these adaptations overall by adaptation classification.

##### Personnel

Personnel changes were related to expanding the staff recruiting caregivers to the intervention, either within the CSP or with staff from outside service lines for the purpose of increasing enrollment. Most adaptations related to personnel were made during implementation of the caregiver classes (*N* = 7) and in response to organizational or setting challenges, such as staff turnover, staff shortages, staff changing position inside or outside the CSP, high workload, and low enrollment of caregivers. Some personnel adaptations (*N* = 5) made during pre‐implementation were planned and intended to give more staff the opportunity to recruit and facilitate the intervention.

“[The change] was made based on experience in recruiting within the CSP program. It gives all CSP staff the opportunity to recruit.” (site 18)

Several sites desired service line collaboration beyond the CSP program for staff to recruit caregivers or co‐facilitate delivering the intervention. However, further into implementation, these sites reported problems with this strategy, as other service lines did not continue to collaborate due to staff workload and shortages or a lack of interest among individual staff.

“Social workers (outside of CSP) and Interns/Fellows have not been recruiting as facilitators due to lack of interest.” (Site 7)

##### Format

Format changes by sites were often “stacking” classes (offering them back‐to‐back), spreading out classes to once per week, offering optional content, and providing classes virtually. These changes in format were most often done to either optimize caregiver participation and engagement or to improve perceived effectiveness by ensuring caregivers took in the information.

“Noted that classes often take longer than 1 hour so this seemed like a good way to balance the content.” (Site 15).

Two sites offered additional class modules. One site decided in the pre‐implementation phase that additional modules responded to the needs of their caregiver population. The other site decided far into implementation to include additional material for continued meetings, conversation, and relationship building based on caregiver feedback.

“There was good communication and relationships with the caregivers who completed the 4 Caregivers FIRST sessions with request to continue. Monthly follow up was decided and booster slides have been utilized.” (Site 20)

Facility leaders at one site required virtual classes due to organizational restrictions on visitors. Lastly, one site applied a strategy from prior implementation experience to recruitment.

“It was in response to a community of practice call that discussed sending postcards to caregivers. We got a great response when we sent them for the support group that we decided we would do them for Caregivers FIRST too.” (site 9)

##### Training

Educating staff within and outside the CSP was an activity some coordinators performed either in‐person during staff meetings or over email. This was done during implementation, with the goal to reach more caregivers by increasing recruitment efforts.

“I've been meeting with other VA programs and educating them on this group along with other supports through the Caregiver Programs.” (Site 4)

There was also a concern in engaging and training other staff for the purpose of assisting with the delivery of the intervention.

“[I have been] training new CSP staff to help with increasing number of staff who can help with facilitating Caregivers FIRST classes.” (Site 8)

##### Content

Changes in intervention content included adding additional sessions, including Spanish materials, replacing standard content with site‐specific tailored content or resources, and removing content felt to not meet communication standards. These changes were made by the local CSP, caregiver participants themselves with staff, and local facility Public Affairs offices. The common goal for these changes were to improve fit with recipients, as well as meet facility communication standards, and address cultural factors. Content was edited for sociopolitical and spoken language/cultural norms, specifically to better align with local communication standards and address regional differences in language.

“Spanish material reviewed and added any appropriate verbiage changes to address regional differences in language.” (Site 20)

“Per Public Affairs Specialist, content that was not aligned with the VA communication standards were eliminated. Additionally, there were some embedded links that were not functioning and needed updating.” (Site 7)

##### Population

Adaptations to the target population occurred twice, and both in high‐touch sites. One site expanded their target population, recognizing the opportunity to reach more caregivers within their CSP by using a newsletter for caregivers and email distribution list. Another site narrowed their population to only those enrolled in the CSP after facing organizational policy requiring additional documentation through another service line, which complicated recruitment efforts.

### 3.3 Relationship between implementation strategy, site characteristics, and types of adaptations employed

Sites with high‐touch implementation support had lower complexity (33.3% vs. 62.5%) and program demand (360 vs. 331 applications), as well as higher staff (11.4 vs. 10.6 people), and organizational readiness at baseline (4.6 vs. 4.3) than low‐touch sites. Rurality, organizational resilience, and implementation climate were similar (see Table 3).

**TABLE 3:** Implementation strategy, site characteristics, and types of adaptations employed.

	Overall (*N* = 20)	Amount of adaptation		Diversity of adaptation	
		One or no adaptations (*N* = 9)	Two or more adaptations (*N* = 11)	One or less classifications of adaptations (*N* = 13)	Two or more classifications of adaptations (*N* = 7)
Full sample (*N* = 20)					
	*N* (%)	*N* (%)	*N* (%)	*N* (%)	*N* (%)
Complexity two or higher	9 (45)	5 (55.6)	4 (36.4)	7 (53.8)	2 (28.6)
Received high‐touch implementation support	12 (60)	5 (55.6)	7 (63.6)	8 (61.5)	4 (57.1)
Rural	4 (20)	2 (22.2)	2 (18.2)	3 (23.1)	1 (14.3)

	Mean (SD)	Mean (SD)	Mean (SD)	Mean (SD)	Mean (SD)
CSP staffing	11 (4)	10 (3)	12 (5)	11 (4)	11 (4)
PCAFC Unique Applications	342.7 (247.6)	355.3 (322.9)	332.3 (197.2)	368.4 (309.5)	294.9 (92.9)
Organizational readiness (baseline)	4.5 (0.3)	4.5 (0.4)	4.5 (0.3)	4.5 (0.4)	4.4 (0.3)
Organizational resilience (baseline)	24.5 (3.1)	24.5 (2.0)	24.4 (3.9)	24.3 (3.0)	24.7 (3.7)
Implementation climate (baseline)	2.0 (0.5)	2.1 (0.6)	2.0 (0.5)	2.1 (0.5)	1.9 (0.6)

High‐touch implementation support sites (*N* = 12)					
	*N* (%) *N* = 12	*N* (%) *N* = 5	*N* (%) *N* = 7	*N* (%) *N* = 8	*N* (%) *N* = 4
Complexity two or higher	4 (33.3)	2 (40.0)	2 (28.6)	3 (37.5)	1 (25.0)
Rural	2 (16.7)	1 (20.0)	1 (14.3)	2 (25.0)	0 (0.0)

	Mean (SD)	Mean (SD)	Mean (SD)	Mean (SD)	Mean (SD)
CSP staffing	11.4 (4.5)	10.6 (4.6)	12.0 (4.8)	11.2 (5.1)	11.8 (3.8)
PCAFC Unique Applications	331.2 (285.5)	363.6 (392.6)	308.0 (212.6)	351.9 (354.0)	289.8 (55.7)
Organizational readiness (baseline)	4.6 (0.2)	4.6 (0.3)	4.6 (0.2)	4.6 (0.3)	4.5 (0.2)
Organizational resilience (baseline)	24.5 (3.5)	24.6 (2.3)	24.4 (4.3)	24.3 (3.6)	24.7 (3.6)
Implementation climate (baseline)	2.1 (0.4)	2.3 (0.3)	2.0 (0.3)	3.1 (0.4)	3.0 (0.2)

Low‐touch implementation support sites (*N* = 8)					
	*N* (%) *N* = 8	*N* (%) *N* = 4	*N* (%) *N* = 4	*N* (%) *N* = 5	*N* (%) *N* = 3
Complexity two or higher	5 (62.5)	3 (75.0)	2 (50.0)	4 (80.0)	1 (33.3)
Rural	2 (25.0)	1 (25.0)	1 (25.0)	1 (20.0)	1 (33.3)

	Mean (SD)	Mean (SD)	Mean (SD)	Mean (SD)	Mean (SD)
CSP staffing	10.6 (3.8)	9.8 (1.2)	11.5 (5.5)	11.4 (3.8)	9.3 (4.2)
PCAFC Unique Applications	359.9 (215.4)	345.0 (268.7)	374.8 (188.3)	394.8 (258.0)	301.7 (145.3)
Organizational readiness (baseline)	4.3 (0.4)	4.3 (0.5)	4.3 (0.2)	4.3 (0.5)	4.3 (0.3)
Organizational resilience (baseline)	24.5 (2.8)	24.5 (2.0)	24.5 (3.7)	24.3 (1.8)	24.7 (4.6)
Implementation climate (baseline)	2.0 (0.7)	2.9 (0.7)	3.0 (0.7)	2.9 (0.6)	3.1 (0.8)

Abbreviations: CSP, Caregiver Support Program; ICS, Implementation Climate Scale; ORIC, Organizational Readiness for Implementing Change; PCAFC, Program of Comprehensive Assistance for Family Caregivers; SD, standard deviation.

#### 3.3.1 High‐touch implementation support

Of the sites with high‐touch implementation support, those who made multiple adaptations—two or more—were more likely to have more staff and lower program demand than sites with one or less adaptations. Additionally, those who made more diverse adaptations—in two or more FRAME classifications—were less likely to be from a facility with high complexity, high program demand, or from a rural area and were slightly more likely to have more staff. Organizational readiness, resilience, and implementation climate did not appear to influence adaptations at sites receiving high‐touch support.

#### 3.3.2 Low‐touch implementation support

At sites with low‐touch implementation support, those who performed two or more adaptations or adapted two or more classifications were less likely to be from complex VAMCs (50% vs. 75%; 80% vs. 33.3%, respectively). Program demand and staffing were higher in low‐touch sites that reported multiple adaptations, yet were lower in those who report more diverse (two or more FRAME classifications) adaptations. Rurality and other organizational characteristics did not appear to have an impact on adaptations for these low‐touch sites.

### 3.4 Adaptations related to intervention early adoption

Sites with two or more adaptations (*N* = 11/20) were more likely to adopt the intervention at 6 months compared to sites with one or no adaptations (90.1% vs. 44.4%) (Appendix Table S2). Sites with two or more classifications of adaptation—a higher diversity of adaptations—were more likely to adopt the intervention overall (85.7% vs. 61.5%). Sites with high early adoption (who adopted and held more than eight classes) were less likely to have made adaptations across multiple categories than those with moderate early adoption (who adopted and held 5–8 classes) (28.6% vs. 57.1%), yet more likely than those who did not adopt at all (28.6% vs. 14.3%).

#### 3.4.1 Adaptation classifications by adoption

While the number of sites making personnel adaptations appear similar across adoption groups, there were twice as many adaptations in sites that adopted compared to those who did not (six adaptations for sites with high early adoption and six adaptions for sites with moderate early adoption vs. three adaptations for sites with no adoption) (Table 4). Sites that adopted the intervention more often made changes in personnel recruiting and delivering the intervention, while sites that did not adopt primarily explored collaboration with other service lines for assistance in intervention recruitment and delivery. Training additional personnel, adjusting the pace of class delivery, and modifying content of the intervention were unique to sites that adopted the intervention, with removing material unique to sites with moderate early adoption and adding materials or classes unique to sites with high early adoption. One site expanded their planned population and met early adoption and another site constricted their population due to organizational policy barrier and did not meet adoption criteria.

**TABLE 4:** Adaptations employed by early adoption.

		Intervention not adopted^a^	Intervention adopted^a^	
	Sites implementing adaptations overall (*N* = 20)	<5 classes (six sites)^b^	5–8 classes (six sites)^b^	>8 classes (eight sites)^b^
Amount and Diversity of Adaptations	*N* sites (%)^a^	*N* sites (%)	*N* sites (%)	*N* sites (%)
Two or greater adaptations	11 (55.0)	1 (16.7)	5 (83.3)	5 (62.5)
Two or more classifications	7 (35.0)	1 (16.7)	4 (66.7)	2 (25.0)

Adaptation Classifications	*N* sites (%)^a^	*N* adaptations^b^	*N* sites (%)	*N* adaptations	*N* sites (%)	*N* adaptations	*N* sites (%)	*N* adaptations
Personnel	9 (45.0)	15	3 (50.0)	3	3 (50.0)	6	3 (37.5)	6
Recruitment	6 (30.0)	6	1 (16.7)	1	2 (33.3)	2	3 (37.5)	3
Delivery	6 (30.0)	6	0 (0.0)	0	3 (50.0)	3	3 (37.5)	3
Service Line	3 (15.0)	3	2 (33.3)	2	1 (16.7)	1	0 (0.0)	0
Format	7 (35.0)	10	1 (16.7)	1	4 (66.7)	5	2 (25.0)	4
Delivery	3 (15.0)	4	1 (16.7)	1	1 (16.7)	1	1 (12.5)	2
Pace	6 (30.0)	6	0 (0.0)	0	4 (66.7)	4	2 (25.0)	2
Training	3 (15.0)	3	0 (0.0)	0	2 (33.3)	2	1 (12.5)	1
Content	4 (20.0)	5	0 (0.0)	0	2 (33.3)	2	2 (25.0)	3
Optional material	2 (10.0)	2	0 (0.0)	0	0 (0.0)	0	2 (25.0)	2
Add class	1 (5.0)	1	0 (0.0)	0	0 (0.0)	0	1 (12.5)	1
Remove material	2 (10.0)	2	0 (0.0)	0	2 (33.3)	2	0 (0.0)	0
Population	2 (10.0)	2	1 (16.7)	1	1 (16.7)	1	0 (0.0)	0

^a^ Sites may make multiple adaptations within a classification, resulting in the number of sites in subclassifications not adding up to the number of sites making adaptations in a classification. All percentages are calculated using the column site sample size as the denominator (e.g., *N* = 20 sites overall, or *N* = 12 high‐touch implementation sites, or *N* = 8 low‐touch implementation sites).

^b^ Adaptations made in subclassifications of an adaptation classification (either Personnel, Format, Training Content, or Population) all do add or sum up to the classification total.

#### 3.4.2 Description of adaptations in implementation by adoption

Sites which did not adopt the intervention by 6 months either did not report any adaptations or solely reported personnel changes, citing reduced available resources such as staff turnover influencing staff ability to recruit for and deliver the intervention. For sites with moderate early adoption (implemented 5–8 classes within 6 months), adaptations were made primarily during the implementation phase, with a mix of proactive and reactive changes with a goal to increase reach and engagement of caregivers. Lastly, sites with high early adoption (facilitated more than eight classes) were more likely to make adaptations in both the pre‐implementation and implementation phases, noting organization/setting, provider, and recipient factors as reasons for change. Recipient factors included available resources, competing demands, coordinator experience implementing the intervention, and recipient motivation, cultural norms, and languages.

## 4 DISCUSSION

Research‐developed interventions, often complex, requiring specialized resources, and tested with external staff are rarely adopted into busy, real‐world settings without adaptations to match a clinical context or target population. Indeed, we found that 20 VAMCs had a range of adaptation experiences over 6 months during national implementation of Caregivers FIRST. Most sites (80%) reported adapting Caregivers FIRST and the majority reported multiple adaptations (55%).

The most common adaptations involved changes to personnel and program delivery/format. Although standardized reporting of intervention adaptations is rare,^24^ previous research suggests that these are common areas that often need adaptation to facilitate adoption and scale. For example, a team‐based telehealth intervention for improving hypertension control in the VA found that intervention content changes improved accessibility and observed changes to staff roles to ensure fit within existing service lines.^7^ Further, a recent systematic review of evidence‐based obesity intervention adaptations prior to scaling similarly found that the most common adaptation was mode of delivery or format.^25^ Unlike previous research, this study provides rich detail on the spectrum of adaptations, ranging from subtle “tweaks” to substantive changes that should be made with caution to ensure that intervention effectiveness or delivery is not compromised. Future real‐world interventions should proactively plan and report adaptations through structured intervention reporting requirements to continue to evaluate these phenomena.

We described the site characteristics associated with varying levels of adaptation. We did not find support for our hypothesis that sites randomized to high‐touch implementation support would report more adaptations than sites assigned to low‐touch support. While those with high‐touch support did have a higher rate of adaptations, there were similar rates in high‐ and low‐touch arms on two or more adaptation classifications—or diversity of adaptations—performed. Also, we hypothesized that sites with higher medical center complexity, more caregiver program staff, higher organizational readiness, more fertile implementation climate, and lower rurality would have a greater number of adaptations. Across these characteristics, we found mixed support for our hypotheses. Sites with multiple adaptations were more likely to receive high‐touch support, had lower medical center complexity, had more caregiver program staff, and lower program demand.

We did not observe differences in organizational resilience or implementation climate between sites with multiple adaptations and sites with one or no adaptations. These findings are in contrast with a recent publication that found organizational readiness was associated with timing of adoption but not adoption overall.^17^ In other words, sites with higher ORIC scores, on average, were more likely to be early adopters of Caregivers FIRST but in this study, there was no difference among ORIC scores between adopters and non‐adopters. Efforts to understand how organizational characteristics influence the implementation process is part of a larger effort within the field of implementation science to better understand why and how organizations adopt evidence‐based interventions like Caregivers FIRST. There are numerous latent and theoretically sound determinants (e.g., organizational characteristics) that are thought to influence the implementation process, but the development of these measures has outpaced efforts to validate them with implementation outcomes in real‐world contexts.^6^, ^26^

We accelerated this effort to examine these determinants of implementation in a real‐world context by describing the relationship between site‐level intervention adaptation and early adoption of Caregivers FIRST among a cohort of sites that had not yet met implementation benchmarks. We found support for our hypothesis (H3a) that sites reporting adaptations would be more likely to adopt Caregivers FIRST at 6 months than sites with one or no adaptations. Specifically, VAMCs with multiple adaptations were more likely to adopt the intervention by a large margin (90.1% vs. 44.4%). Through qualitative analysis, we found balance between proactive adaptations made prior to implementation with reactive adaptations made during implementation unique to positive deviants (more than eight classes delivered), as well as sites considering recipient factors (i.e., motivation/readiness) for adaptations made. Service line collaboration and narrowing the caregiver population were primarily seen in sites that did not adopt the intervention. It is possible these adaptations take longer to make an effect on recruitment and delivery, delaying adoption, or are more difficult to achieve. These findings are novel and warrant further research.

### 4.1 Limitations

There are several limitations to this study. First, sites randomized to receive only low‐touch implementation support had some differences in characteristics (geographic region, facility complexity, and experience implementing evidence‐based practices) which may have influenced their ability to be early adopters in ways we do not detect. Second, we undertook extensive systematic data collection to document adaptations by site, however, we may have missed some unplanned adaptations or adaptations inconsistent with fidelity that sites may have acted upon using our survey due to its one‐time delivery during implementation of Caregivers FIRST. Sites randomized to high‐touch implementation support did have opportunities to consider and report adaptations during enhanced support team calls and this may partially explain the higher number of adaptations among sites receiving high‐touch support. Future research on these conversations, the prevalence of unplanned adaptations in reaction to unanticipated challenges, and their impact on intervention adoption or non‐adoption should be explored. Lastly, this study occurred in an integrated healthcare system with a shared EHR and therefore high quality and standardized reporting of implementation activities (including adoption) across sites may not be possible in other systems in the United States.

Finally, given the few reports of caregiver training programs that had been implemented nationally, we are unable to discern whether the early adoption rates observed in this paper are high or low. We also do not yet know as of this writing the final adoption rates for the overall study by intervention arm nor by adaptations made (primary outcome is “penetration at 12 months”).^12^ Furthermore, not all caregivers may desire the training or feel comfortable engaging in a group setting. Some may be currently engaged in other CSP services than Caregivers FIRST (e.g., peer mentoring or individual counseling) or desired to participate but were unable to afford the time or cost to do so.

To remove caregiver engagement barriers, health systems could consider a combination of strategies, such as reimbursing for gas, “prescribing” the training as a needed service, including cash incentives, providing care assistance for the duration of the training, or conducting the trainings outside of working hours to allow working caregivers to attend. Such changes could also increase equity, along with standardized identification in the electronic health record^27^ so that providers could target trainings to all caregivers most likely to benefit. Along with these ideas, health systems may want to create processes detailing the scope of allowable adaptations for caregiver trainings based on the staff capacity and the individual caregivers' training needs,^28^ in order to obtain sustained success of programs like Caregivers FIRST.

### 4.2 Implications

FRAME was central to reporting and classifying adaptations. Site‐level adaptations varied by number and diversity. At 6 months, VAMCs with two or more adaptations adopted Caregivers FIRST at more than twice the rate than VAMCs with fewer adaptations. Understanding the role of adaptation in adoption success could assist other healthcare systems to implement interventions supporting caregivers. Increasing pragmatism of trainings, including through adaptation, is more important than ever given the increasing incentivizing of expanding group caregiver trainings nationally in and outside VA. The Centers for Medicare and Medicaid Services for the first time in 2024 began allowing providers to bill for caregiver group trainings.^29^, ^30^ As such, as health systems search to identify pragmatic trainings to offer, the results in this paper could be useful. The proactive tracking of adaptation that we undertook is novel and can facilitate an awareness of adaptation, ensuring that fidelity of evidence‐based training components remains. Future research should identify best practices of supporting and monitoring intervention adaptation, including the role of adaptation in sustainment.

## SPONSOR'S ROLE

The funding agency had no role in the design or conduct of the study; collection, analysis, or interpretation of the data; or preparation, review, or approval of the manuscript. The contents do not necessarily represent the views of the U.S. Department of Veterans Affairs or the US Government.

## AUTHOR CONTRIBUTIONS

Conceptualization, A.B., C.D., L.L.Z., J.H.; Funding acquisition, S.N.H., K.D.A., and C.H.V.H.; Project coordination, K.D.; Data analysis, A.B., K.D., C.D., S.K., C.J.C.; Writing—original draft, A.B., C.D., C.H.V.H., K.D., and L.L.Z; Writing—review and editing, T.C., N.R.S., M.S.B., E.F., S.K., C.J.C., S. N.H., and K.D.A. All authors read and approved the final manuscript.

## FUNDING INFORMATION

This work was funded by the United States (U.S.) Department of Veterans Affairs Quality Enhancement Research Initiative (QUE‐20‐023), the VA Caregiver Support Program, and the Center of Innovation to Accelerate Discovery and Practice Transformation at the Durham VA Health Care System (CIN 13‐410). Courtney Van Houtven and Kelli Allen are supported by the U.S. Department of Veterans Affairs, Veterans Health Administration, Office of Research and Development, Research Career Scientist Program (RCS‐21‐137 and RCS‐19‐332). Amanda Blok and Megan Shepherd‐Banigan are supported by U.S. Department of Veterans Affairs, Veterans Health Administration, Office of Research and Development, Career Development Award (CDA 21‐161 and CDA 17‐006). The funding agency had no role in the design or conduct of the study; collection, analysis, or interpretation of the data; or preparation, review, or approval of the manuscript. The contents do not represent the views of the U.S. Department of Veterans Affairs or the US Government.

## CONFLICT OF INTEREST STATEMENT

The authors declare no conflict of interest.

## Supporting information


**Data S1. Figure S1.** Adaptation Survey Consort.
**Table S1.** Baseline site characteristics by implementation arm.
**Table S2.** Types of Adaptations Employed by Early Adoption of Intervention.
