# Supplementary material for: Adaptations and early adoption of a family caregiver intervention in the Veterans Affairs Health Care System: A multimethod pragmatic approach for national scaling
Source: Health Serv Res. 2024 Aug 1;59(Suppl 2):e14360. doi: 10.1111/1475-6773.14360 (PMC11540588; doi:10.1111/1475-6773.14360)
Supplement: Supplementary file 1 — Data S1. Figure S1. Adaptation Survey Consort. Table S1. Baseline site characteristics by implementation arm. Table S2. Types of Adaptations Employed by Early Adoption of Intervention. [file HESR-59-0-s001.docx]

**Appendix Figure 1. Adaptation Survey Consort**


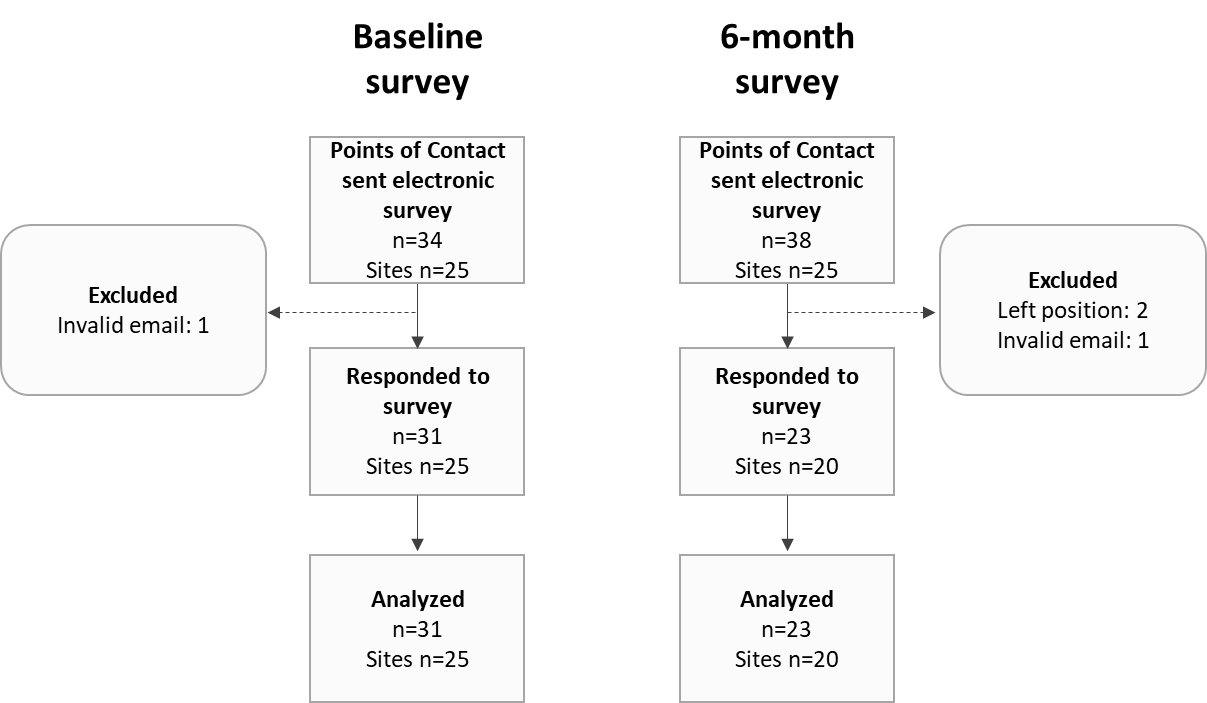


| **Appendix Table 1. Baseline site characteristics by implementation arm** | | | |
| --- | --- | --- | --- |
|  | **Overall** (N=25) | **Implementation Strategy Arm** | |
|  |  | Low-touch  (n=12) | High-touch  (n=13) |
|  | N (%) | N (%) | N (%) |
| Facility complexity, 2 or higher | 13 (52) | 8 (67) | 5 (38) |
| Rural | 5 (20) | 3 (25) | 2 (15) |
| Location |  |  |  |
| South | 10 (40) | 7 (58) | 3 (23) |
| Northeast | 7 (28) | 3 (25) | 4 (31) |
| Midwest | 5 (20) | 1 (8) | 4 (31) |
| West | 3 (12) | 1 (8) | 2 (15) |
| Site’s experience implementing evidence-based practices^a^ |  |  |  |
| Quite a lot, a fair bit, or some | 22 (88) | 12 (100) | 10 (77) |
| Very little or none | 0 (0) | 0 (0) | 0 (0) |
| Don’t know/Missing | 3 (12) | 0 (0) | 3 (23) |
|  | Mean (SD) | Mean (SD) | Mean (SD) |
| CSP staffing | 11 (4) | 10 (3) | 12 (4) |
| PCAFC unique applications | 358 (232) | 382 (190) | 336 (263) |
| Organizational readiness | 4.5 (0.3) | 4.4 (0.3) | 4.6 (0.2) |
| Organizational resilience | 24.5 (2.9) | 24.3 (2.4) | 24.7 (3.3) |
| Implementation climate | 2.1 (0.5) | 2.1 (0.6) | 2.1 (0.4) |
| *Abbreviations:* *SD, standard deviation; CSP, Caregiver Support Program; PCAFC, Program of Comprehensive Assistance for Family Caregivers; ORIC, Organizational Readiness for Implementing Change; ICS, Implementation Climate Scale.*  *^a^ Facility experience implementing evidence-based practices is self-reported and was collected by each site point of contact at study intake.* | | | |

| **Appendix Table 2. Types of Adaptations Employed by Early Adoption of Intervention** | | | | | |
| --- | --- | --- | --- | --- | --- |
|  | Overall (N=20) | Amount of Adaptation | | Diversity of Adaptation | |
|  |  | 1 or no adaptations (N=9) | 2 or more adaptations (N=11) | 1 or less classifications of adaptations (N=13) | 2 or more classifications of adaptations (N=7) |
|  | N (%) | N (%) | N (%) | N (%) | N (%) |
| Adopted intervention at 6 months | 14 (70.0) | 4 (44.4) | 10 (90.1) | 8 (61.5) | 6 (85.7) |
| Adopted intervention; >8 classes | 8 (40.0) | 3 (33.3) | 5 (45.4) | 6 (46.2) | 2 (28.6) |
| Adopted intervention; 5-8 classes | 6 (30.0) | 1 (11.1) | 5 (45.4) | 2 (15.4) | 4 (57.1) |
| Intervention not adopted at 6 months | 6 (30.0) | 5 (55.6) | 1 (9.1) | 5 (38.5) | 1 (14.3) |
